# Paleoenvironmental changes in river channel systems in alpine rockslide deposits exemplified by the Fernpass rockslide in the Tyrolian Außerfern District, Austria

**DOI:** 10.1038/s41598-022-25922-8

**Published:** 2022-12-23

**Authors:** Christian Wolkersdorfer

**Affiliations:** 1 SARChI Chair for Mine Water Management, Department of Environmental, Water and Earth Sciences, Tshwane University of Technology, Private Bag X680, Pretoria, 0001 South Africa; 2 Weidach 16, 6632 Ehrwald, Austria

**Keywords:** Environmental sciences, Hydrology, Hydrology

## Abstract

This paper describes the instability of river channel systems in alpine rockslide deposits using the Fernpass Rockslide and the river Loisach in the Tyrolian Außerfern District (Austria) as an example of paleoenvironmental developments. This is the first investigation of this kind of the Fernpass, one of the most important Alpine north–south transport connections since the bronze age. It uses geomorphological, sedimentological, onomastic and hydrogeological investigations to reconstruct the course of a late Holocene river in this area and a probabilistic simulation for dating. Tracer tests assisted in investigating the potential groundwater connections of the river systems. The findings show that the Palaeoloisach runs on the orographically right side in a marginal valley of the Fernpass furrow and changes to the orographically left side of the furrow within the Rauth suburb in the village of Biberwier. A probabilistic simulation of the Narrenbichl slip event, which changed the course of the Palaeoloisach, dates the event to an age of 664 ± 116 BC. This investigation is an important contribution to understanding Quaternary postrockslide developments, how groundwater contributes to forming postrockslide channel systems and archaeological findings occurring in populated areas.

## Introduction

Both, internationally and in the Alps, the instabilities of river channels in rockslide deposits resulting from paleoenvironmental changes have not been the focus of research in recent years. Most of the research has investigated the effect of rockslides on rivers or lakes and their erosion history or climatic effects for rockslides to occur, but not their post-rockslide development or groundwater–surface water interaction^1–9^. This is, therefore, a first attempt to use the course of a palaeoriver (Palaeoloisach) in the north-eastern Außerfern District, Tyrol/Austria, around the northern branch of the Fernpass rockslide (47° 22′ 29″ N 10° 52′ 49″ E, WGS84), as a case study for post rockslide environmental development and its implications for historical developments and groundwater flow. The Fernpass is one of the most important north–south connections in the Alps, has been used without interruption since bronze age times^10^ and was once an important Roman road name *Via Claudia Augusta*^11,12^. While the general geomorphological and geological conditions of the rockslide have been described in previous studies^13,14^, to date, no publication has addressed the paleoenvironmental, hydrological or hydrogeological conditions in the headwaters of the Loisach River and the development of channels draining the Fernpass rockslide area towards the east into the Außerfern District. In addition, no airborne geophysical surveys, as exemplified for paleochannels in Australia^15^, to identify this type of geological feature have been conducted there. Therefore, this study aims to close a gap in hydrogeological research for the geological and paleoenvironmental conditions in the Außerfern, and a gap in investigating post hydrological developments in large rockslides in general. This study contributes to the understanding of the postglacial development of the Fernpass furrow and especially its history after the Fernpass rockslide.

For the Bavarian part of the river Loisach or the close by river Partnach in the Wetterstein Mountains, numerous studies are available about the post- and preglacial Loisach, its sediments and paleocourse and the hydrology of the Wetterstein Mountains as well as the groundwater conditions in these areas^16–20^. Also the effects of the Eibsee rockslides on the paleolake Eibsee have been studied in detail^8,9^. So far, the only studies dealing with the hydrogeological conditions in the Außerfern part of the Loisach are unpublished and contain no detailed description of the river or its past course^21–23^. *Palaeoloisach* (in German: *Paläoloisach*) is chosen as a terminology to distinguish it from *Urloisach*, an older glacial stream channel or palaeoriver that exists in Bavaria. The latter is an interglacial river course^24^, which was first described by Knauer^16^ and studied in more detail by Meyer and Schmidt-Kaler^25^. As the Fernpass rockslide occurred postglacial, approximately 4100 years ago^26–28^, the age of the Tyrolian Palaeoloisach must be younger than that of the Bavarian Urloisach. However, the Palaeoloisach must not be understood as a potential course of the Loisach before the Fernpass rockslide. Detailed studies about Loisach’s pre-rockslide course are still missing, although pre-rockslide drainage to the south into the Inn Valley has been repeatedly discussed in the literature^29–31^. This course of the Loisach should be called “Pre-Loisach”, and the term “palaeoriver” means an “ancient river […] that once occupied fluvial palaeovalleys […], or which deposited ancient basinal fluvial successions […]”^32^.

**Figure 1:**
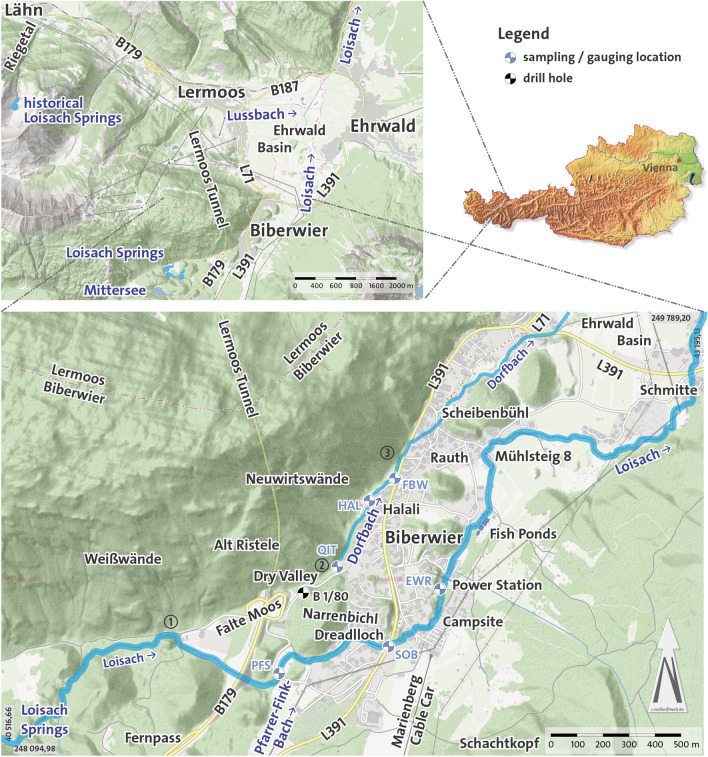
Maps of the study area with the toponyms mentioned in the text. Three letter codes refer to sampling/gauging stations; QIT: Quelle im Tal, HAL: Hotel Halali, FWB: Feuerwehr Biberwier, PFS: Pfarrer Fink Steig, SOB: Hotel Sonnenbichl, EWR: Elektrizitätswerke Reutte (modified tirisMaps data as of 2020-01-21: https://maps.tirol.gv.at, coordinate system: Gauß-Krüger M28–MGI Austria GK West/EPSG 31,254).

In this paper, an area ranging from west of Biberwier, now known as the Loisach springs (Loisachquellen), to the entrance of the Loisach (Austrian river code 2-6-26) into the Ehrwald Basin (also referred to as “Lermoos Moos”; Fig. 1) at local road L391 (Ehrwalder Straße, Schmitte suburb) is covered. In addition to the Loisach River, the area is traversed by the Dorfbach rivulet (village brook, 2-6-26-3a), which flows into the Loisach 400 m north of the Schmitte suburb, both flowing essentially in a SW–NE direction. In the Ehrwald Basin itself, the course of the Loisach has been strongly altered by river regulation since the end of the nineteenth century^33,34^. Since 2014, the dead stream branches of the Loisach have been part of the “Ehrwalder Basin Nature Reserve”, which was declared a nature reserve in 1991^35^ and ensures long-lasting protection of its bogland vegetation^36^.

## Methods

While investigating the hydrogeological situation in the northern branch of the Fernpass rockslide, between the Loisach springs and the village of Biberwier, several dry valleys were identified, suggesting that the Loisach, compared to today, might have had a different post Fernpass rockslide course. The geological map 1:50,000 (GEOFAST 116—Telfs, edition 2011/04), Lidar data (Airborne Laserscanning data: TirisMaps), small-scale geological mapping and field investigations, core drillings, three small-scale tracer tests, hydrogeochemical analysis (main ions and trace elements) and historical maps (Tiris Historische Karten Tirol), especially the onomastic section, were used for this investigation. Furthermore, results from the literature were used to verify the findings in the field. In addition, observations of the locals in Biberwier about Loisach and Dorfbach were included to evaluate the on-site conditions. pH, water temperature, and electrical conductivity were measured on site with WTW portable instruments. All samples, which were kept in cooler boxes or fridges until sampling, were filtered through 0.45 μm cellulose acetate filters, and for the trace element analyses, HNO_3_ was added. Water samples were analysed with a Perkin-Elmer AAS-3300 Spectrometer for zinc, sodium, potassium, calcium, and magnesium and DIONEX-IC-DX-100 for fluoride, chloride, nitrate, and sulphate. Flow was measured with a Sommer (Sommer GesmbH, Vorarlberg, Austria) salt dilution measurement system at six locations (Fig. 1). Tracers used for this study were uranine (sodium fluorescein), and they were detected with a GGUN (Pierre Schnegg, Neuchâtel) field fluorimeter at 4 min sampling intervals. To date the Narrenbichl slip event, a probabilistic approach with the Monte Carlo method and Simulación 5.0.5 (José Ricardo Varela, 2020: https://sites.google.com/view/simulacion5) was applied. After ten thousand simulation steps with nine input parameter variables and one output variable the results were considered statistically significant. All parameters were simulated within the range of their natural variability, and their distribution was modelled with Simulación’s build in “Fit distribution to data” tool.

### Conference presentation

This paper is a substantially extended and modified version of a preliminary study presented in German language at the Geoforum Umhausen (Tyrol/Austria) conference^37^.

## Onomastic investigation about the name Loisach and its springs

In addition to the hydro(geo)logical and environmental change of the Loisach, the river has undergone a name change. It is therefore important to understand this historical process to avoid confusion with the river’s name and location. This section summarizes the onomastic knowledge and tries to identify the reasons for the confusion.

During the past 450 years, the spring of the river “Loisach” was located at various places, not always in the Fernpass area, where it is located today^38,39^. Additionally, the name of the river changed over the centuries (Table 1), which has been discussed in more detail by Stolz^40^ and Reitzenstein^39^. No Roman name for the river has been handed down. Several authors already described the location of the spring, such as Weber^41^: “The origin of this major part of the Bavarian River can be found at Lähn. The other tributaries of all the mountains that are collected at Ehrwald descend northwards under the Ehrwalderschanze, leave the country, and after a mostly northeastern course of 8 ½ miles flows into the Isar near Wolfratshausen”. Nearly identically, Schaubach^42^ writes, “From Lermos we follow the post road to Lähn, where the Loisach rises, which we already got to know when describing Reutte”. Borne^43^, similarly, describes: “The Loisach rises in Tyrol between Reutte and Lermoos in Zwischenthoren, flows in the calcerouse Alps over Lermoos and Garmisch, enters Eocänschichten at Eschenlohe, flows through Lake Kochelsee here, the swampy Rohrsee in its northeastern continuation and the Haselmoos enclosing these lakes, and enters the Schwäbisch-Bavarian Plateau at Rain”. Müller^44^ wrote 24 years later: “Behind the hermitage, the road leads through the high, richly debris-covered Hinterthorn valley via Heiterwang (991 m) and Bichelbach to the often avalanche stricken Lähn, a place not far from the Loisach spring, and then descends to Lermoos (989 m) into the uppermost valley basin of this river, which today is probably richly debris-covered, but whose moorland clearly enough points to the earlier water cover of the remarkably large basin”. Concerning the meaning of the name Loisach, Reitzenstein^39^ and Greule and Hackl-Rößler^45^ deduce that it can be considered the “pleasant stream”.

**Table 1:** Historical names of the rivers now known as Loisach and Lussbach in historical maps of Tyrol and in the literature.

Year	Loisach	Lussbach	Reference
Eighth century	Liubisaha (outside mountains)	–	Widmoser 1970
1470	Leussach (at Grießen pass)	–	Widmoser 1970
1530	Ach zue Lermos (possibly includes Lussbach)	–	Widmoser 1970
1561	– |Fons Loyse^b,e^	–	Lazius 1561
1573	– |Loÿsa fluss^b,e^	–	Ortelius 1573
1595	Loik, fons|Loisa Fluss^b,e^	–	Bertellus 1595
1604	– |Loÿsa fluss^b,e^	–	Warmund Ygl 1604
1609	die Loÿsa	–	Burglechner 1609
1654	Loÿsa fluss	–	Sanson 1654
1662	Loisa fluss	–	Hurtero 1662
1749	Ach zu Biberwier	–	Widmoser 1970
1760	Achen Bach|Laysach (Loysach Bach)^e^	Rigl Bach	Anich and Hueber
1765	Achen-Bach^c^	–	Peter Anich
1768	Gemeine Ach		Widmoser 1970
1798	–	Loysach	Bacler d’Albe
1810	Aach Bach	Loisach Bach	‘1. Landesaufnahme’
1861	Louisach Bach	Riegel Bach	Kulturen-Skelett
1870	– |Ach|Loisach	Riegl Bach	‘2. Landesaufnahme’
1872	–	Loisach Bach	
1881	–	Loisach	Borne 1881
Nineteenth century	–	Lauchwaldbach, Loisach, Lus	Moser 1932
Nineteenth century	– |Loisach^d^	–	Spezialkarte 1:75.000
1904	Achen Bach|Loÿsach Bach		‘Straßenkarte’
2015	Schmittebach|Loisach^a^	Rigltalbach|Lussbach^e^	Katastermappe
2016	Loisach (Feuer-Bach)|Kleine Ach (Loisach)^a^	Rigetal-Bach|Luss-Bach^e^	AV-Karte 4/1 digital
2018	Loisach (2-6-26)	Lussbach (2-6-26-4)	Tiris

Sources in documents and publications are listed in Reitzenstein^39^.

^a^Upstream|downstream Schmitte.

^b^Exact course not clear.

^c^Upstream of the confluence with the Lussbach.

^d^Upstream|downstream of the confluence with the Lussbach.

^e^Upstream|downstream Gries.

Thus, the river that flows through Lermoos today, known as Lussbach, once bore the name Loisach and had its source south of Lähn. As the origin of watercourses is usually at the point furthest away from its mouth, the source of the Loisach should indeed be located there. In fact, the distance from where the Lussbach rises to its confluence with the Loisach is approximately 7.7 km but only 6 km from there to today’s Loisach springs. Therefore, the Loisach spring should be correctly located in the Riege valley near Lähn. However, according to the official Tyrolian Water Book (QU70803002), “[the] spring catchment of the Loisach spring […] lies approx. 650 m west of the water reservoir of the Brünnen spring and approx. 800 m east of Lake Mittersee. It originates at the foot of a wooded, steep slope inclined from west to east; approx. 80 m west a hiking trail leads to lake Mittersee”. This location is in property 1908/2 municipality 70803 Biberwier cadastral municipality 86003 Biberwier (easting: 40456.75 northing: 248193.22 meridian: M28 elevation: 1061.21 masl—meters above Adriatic Sea level), GK100009. In the official water book, the Loisach is still sometimes referred to as “Schmittebach” without the reason being known (e.g., water book T20840289R3).

**Figure 2:**
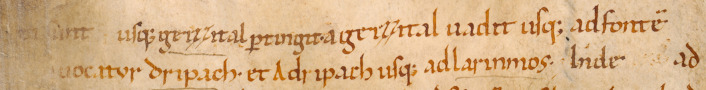
Part of the Freising Urkundenbuch page (*Codex commutationum*—BayHStA HL Freising 3b, vol. 301), where the Loisach springs (“*fontem qui vocatur dripach*”) are first mentioned (urn:nbn:de:bvb:12-bsb00003038-8).

**Figure 3:**
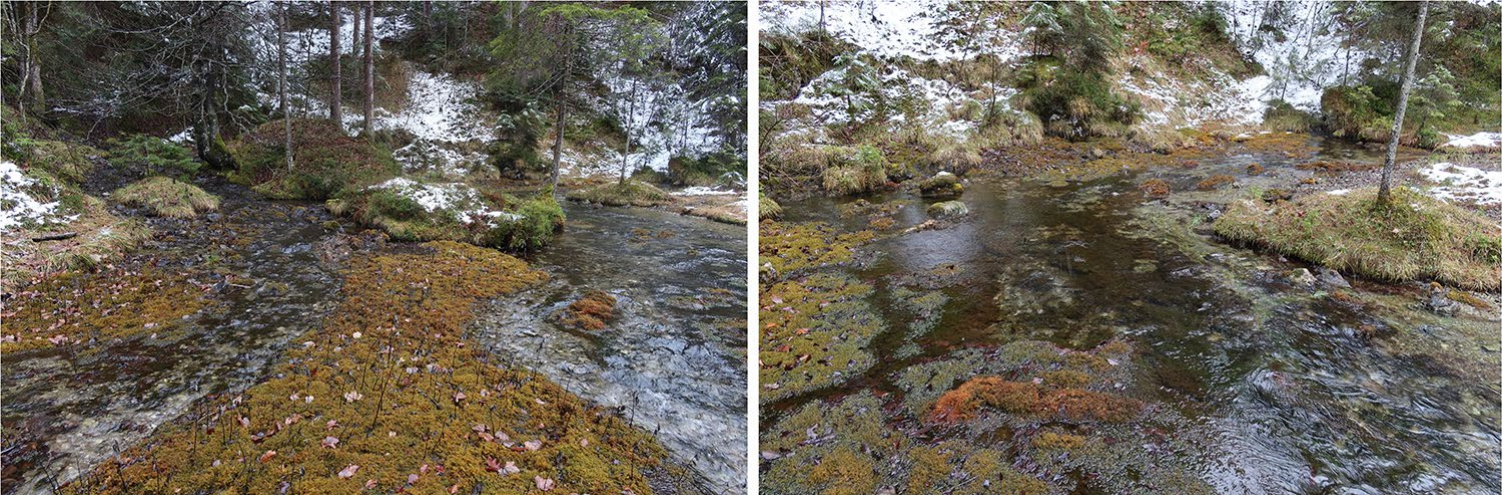
Area of the Loisach Springs SW of Biberwier. Left: Loisach spring s.s. (source area 1); right: source area 2, which can be seen on the right side of the left image (taken: 2019-12-25).

Possibly, the springs are first mentioned in a boarder description of the diocese Freising, dated at approximately 1073–1078 CE^46^. Based on topographical indications, “the springs which are called Dripbach” in the Latin description “… *a geizzital vadit usque; ad* ***fontem qui vocatur dripach*** *et a dripach usque; ad larinmos. …*”^47^ very likely refer to today’s Loisach springs (Fig. 2). “*Dripach*”, based on Old High German language, can be translated with “Three Rivers”^48^, which is an indication of the three Loisach spring areas mentioned below (Fig. 3). Locating the “Three Rivers” near lake Plansee, as suggested by^49^, would contradict the order in which the mediaeval writer listed the names of the boarder’s 21 fix points.

The reasons why the “Loisachquellen” (Loisach springs) today lie southwest of Biberwier and when the naming became generally accepted could not yet be determined. Stolz^40^ mentions that the early cartographers assumed that the river’s sources were lakes, and as Loisach once emerged into a lake in the Ehrwald/Lermooser Moos before its drainage in the nineteenth century^40,50^, this can be considered the reason why Loisach’s course upstream of the former lake is unclear. Interestingly, the names of the Fernpass lakes have not substantially changed since the sixteenth century. In 1536^40^, they are listed as Plinndt See, Mittersee and Weyssen See (today: Blindsee, Mittersee, Weißensee).

## Results and discussion

### Today’s course of the Loisach and the Dorfbach

A Loisach spring *sensu stricto* cannot be localized; rather, it is a semicircled source area with many springs located southwest of Biberwier. From the drinking water intake of the Biberwier municipality to the “Loisachquelle” stamp station, it comprises a large number of smaller and larger spring outlets over a length of 300–400 m (Fig. 3). These can be grouped into three source areas, including a set of springs north of the Biberwier drinking water supply.

**Figure 4:**
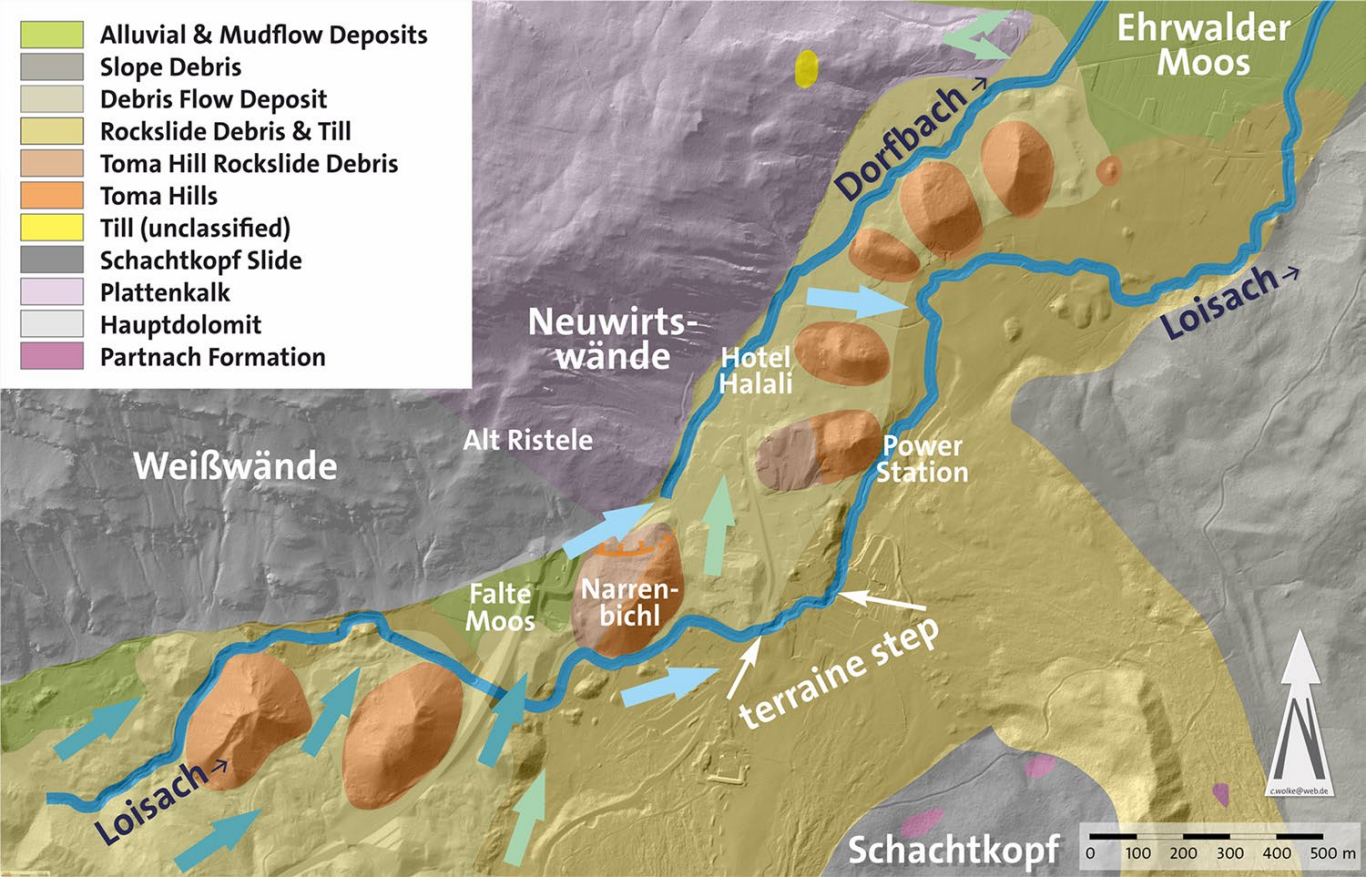
Geological, geomorphological and hydrological overview of the north-western Fernpass area. Dark blue arrows: groundwater flow according to Schuch 1981^23^, light blue arrows: ground water flow identified by this study, green arrows: ground water flow based on Uranine tracer tests (sources: tirisMaps Lidar data: https://maps.tirol.gv.at; Abele 1964^53^; Schuch 1981^23^; Geological Map GEOFAST 116—Telfs, edition 2011/04: © 2011 Geologische Bundesanstalt Wien; own investigations).

**Figure 5:**
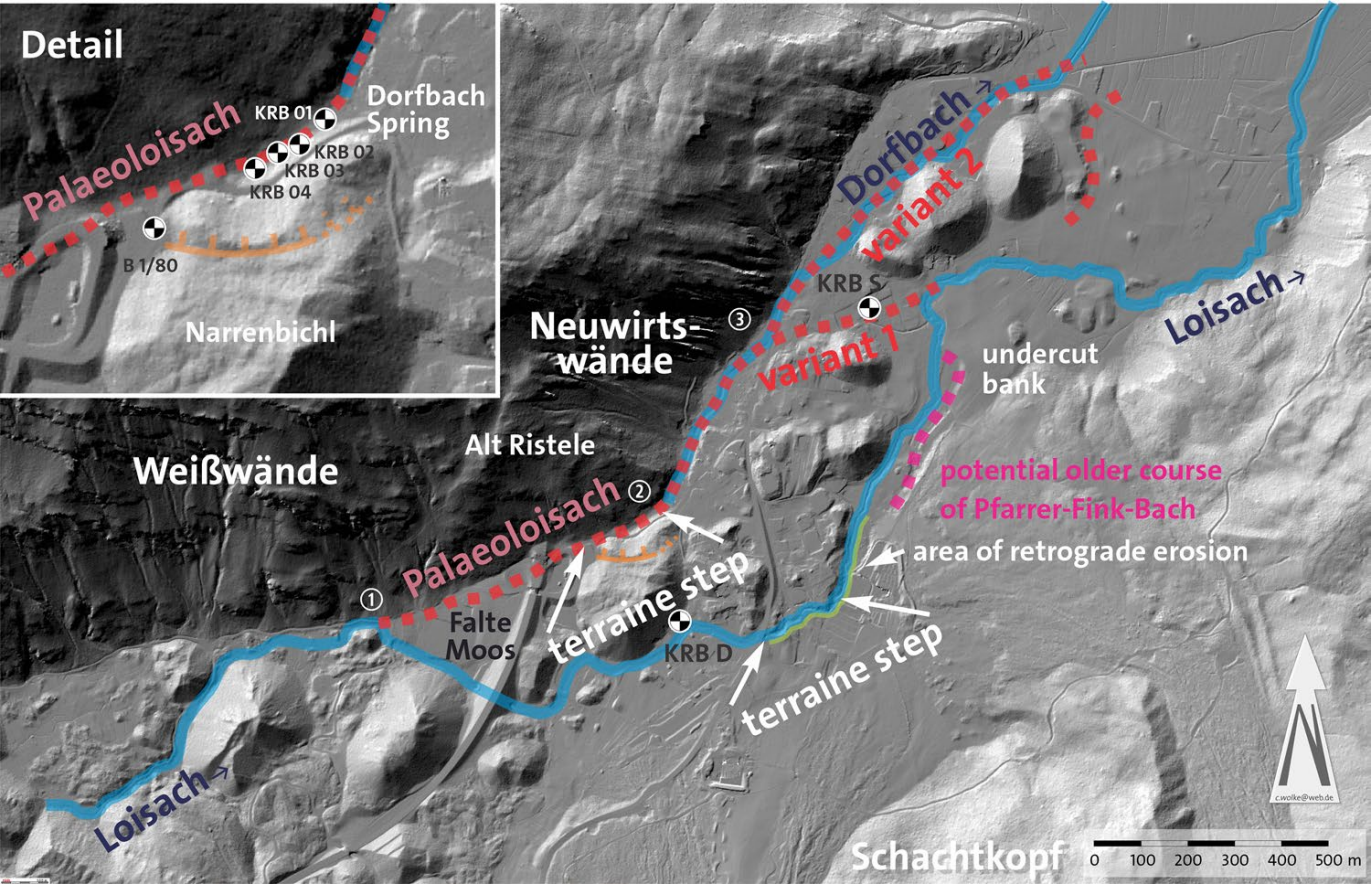
Loisach River and Dorfbach rivulet southwest of Biberwier and postulated course of the Palaeoloisach with variants 1 and 2. The edge in the terrain, indicating the slip of the Narrenbichl, is marked (modified tirisMaps Lidar data: https://maps.tirol.gv.at; software used: PanaVue ImageAssembler 3.6 for stitching the images and CorelDraw 21.3 for text and symbols).

**Figure 6:**
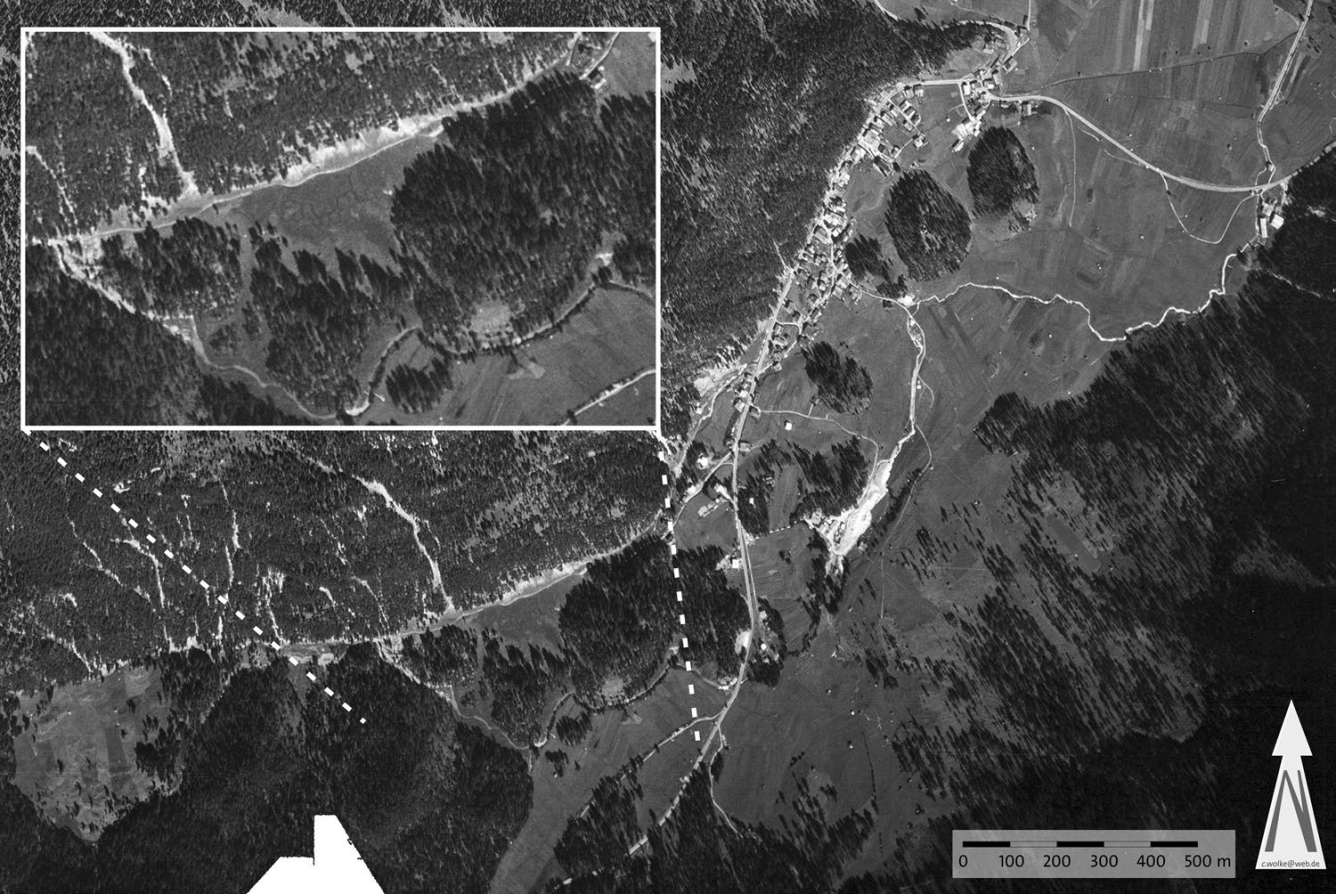
British aerial photograph of Biberwier from August 1953 with a “football field”. The lighting conditions clearly show the course of the Loisach. The wetting zone and the channel running down from Weißwände can be seen in the picture. Names of the features in Fig. 4 (source: NCAP_JARIC_ASM_0070_AMS_13343: NCAP/ncap.org.uk; software used: CorelDraw 21.3).

Starting from the source area at 1061 masl, Loisach initially runs 530 m in a northeasterly direction over Fernpass rockslide debris and tills until it meets the steep drop of Weißwand (Hauptdolomit dolomite, Fig. 4), where it bends and follows that rock formation for 350 m in an easterly direction (Fig. 5). Shortly before the Biberwier sports field (local field name Falte Moos; before the tunnel construction a bogland), it changes its course to southeast for 400 m, and after flowing between two Toma hills and under the Fernpass road (B179), it changes its course to northeast for 170 m. Toma hills (or *Tomas*) are cone shaped hills typical of some rockslide deposits, considered to be a result of internal erosion^51^. Since 1982, the area around the Falte Moos consists predominantly of excavated material from the Lermooser Tunnel^52^, which gives rise to the suspicion that the Loisach had been diverted during the construction work. However, this is not the case, as historical maps show. Rather, before constructing the new Fernpass road bypass, the course of the Loisach was approximately the same as it is today (Figs. 5 and 6). Abele^53^ maps a “sedimentation zone” as well as a “debris flow or alluvial cone” at this location, and on the cadastral map of 1856, a river comes down from Weißwand, which then flows into Loisach near today’s Fernpass road underpass.

The Loisach then flows southeast around Toma Narrenbichl (today commonly known as Sonnbichl) for a length of 260 m and then bends southeast for 170 m towards L391 (1019 masl). During these 1880 m from the headwaters to L391, the Loisach had a gradient of 22.3 ‰.

From there, the Loisach flows for 190 m over a step in the terrain (indicated in Figs. 4 and 5), entailing a difference in height of 15 m (1005 masl), corresponding to a gradient of 78.9 ‰. This elevation of 1005 masl corresponds to the elevation of the Dorfbach spring on property 157/9 (QIT in Fig. 1), south of estate Im Tal 15. Retrograde erosion of the Loisach can clearly be seen at this location, which, however, was stopped by Fernpass road construction.

From this point, the Loisach flows 780 m in a northern direction, passing several Toma hills on their eastern side. The flanks in the alluvial and mudflow cones indicate that over time, the river has moved its bed approximately 70 m to the west. Between the campsite and the Mühlsteig 8 estate, there are bouncing slopes and a small, partly dry valley south of the fishponds, from which numerous smaller springs emerge, supplying several fishponds.

**Figure 7:**
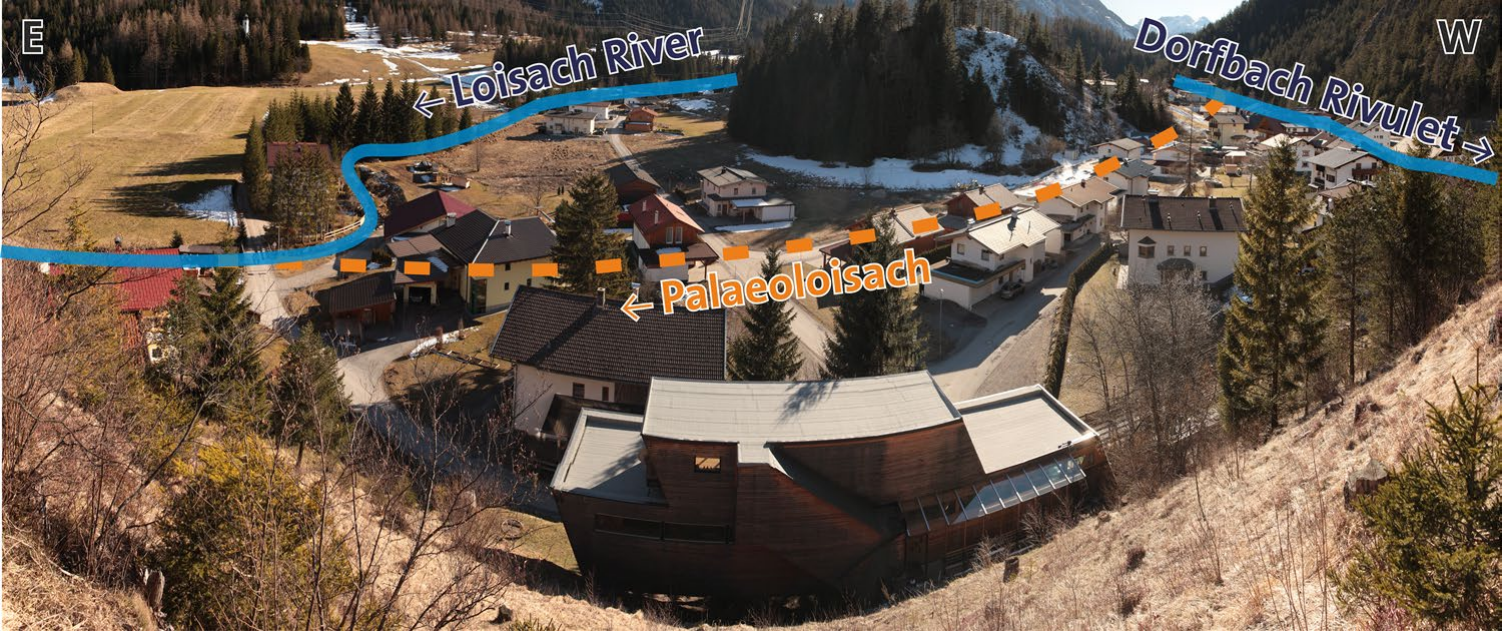
Biberwier, Rauth district, looking to the south (February 2014). On the right, the valley of the Dorfbach rivulet and on the left, at the bridge, the bend of the Loisach River. The valley between the photo location and the Toma hill opposite is filled with fluvial sands and gravel and is drained by a ditch towards the Loisach River. It once hosted the Palaeoloisach.

In the Rauth area, the Loisach meets a transverse Toma, named the Scheibenbühl (Fig. 7) according to Blatt 19 of the 2nd Josephinisch-Franziszeische surveying^54^, and changes its course for 600 m to the east, before entering the Ehrwalder Moos in a northerly direction at 967 masl. Up to there, the Loisach covered a distance of 1780 m and an altitude difference of 38 m from the end of the step in the terrain to the connecting road to Ehrwald, which corresponds to a gradient of 21.3 ‰ and is roughly identical to the gradient above the step in the terrain.

**Figure 8:**
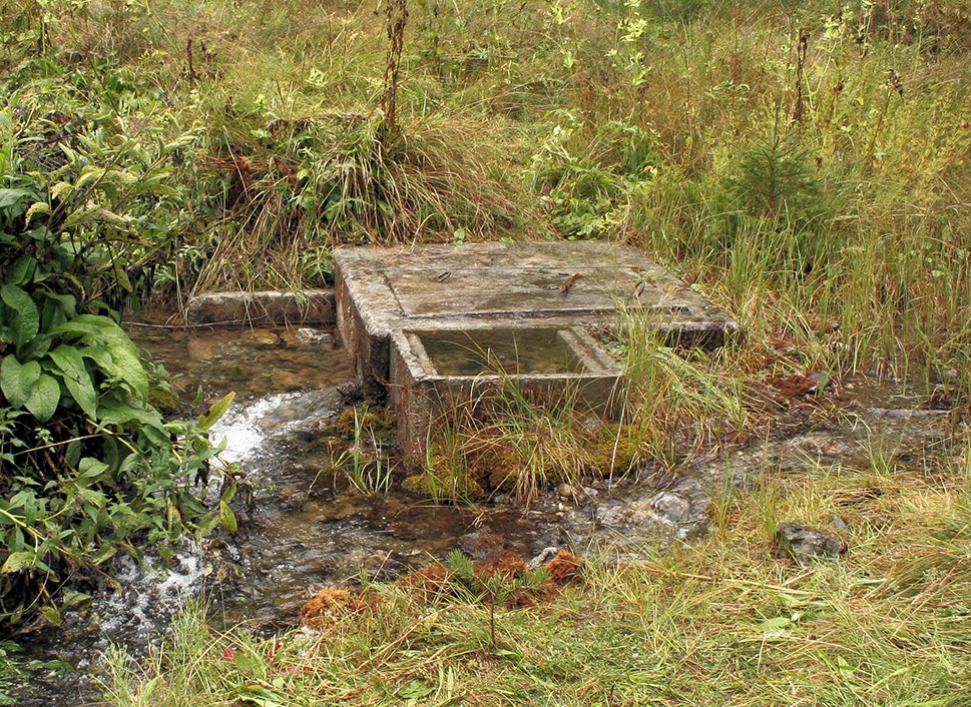
Springs of the Dorfbach. Historically, the water was used as Biberwier’s drinking water supply. The concrete structure captures the middle of the three springs, the other two coming from the left and the right to the structure (image taken 2007-09-06).

**Figure 9:**
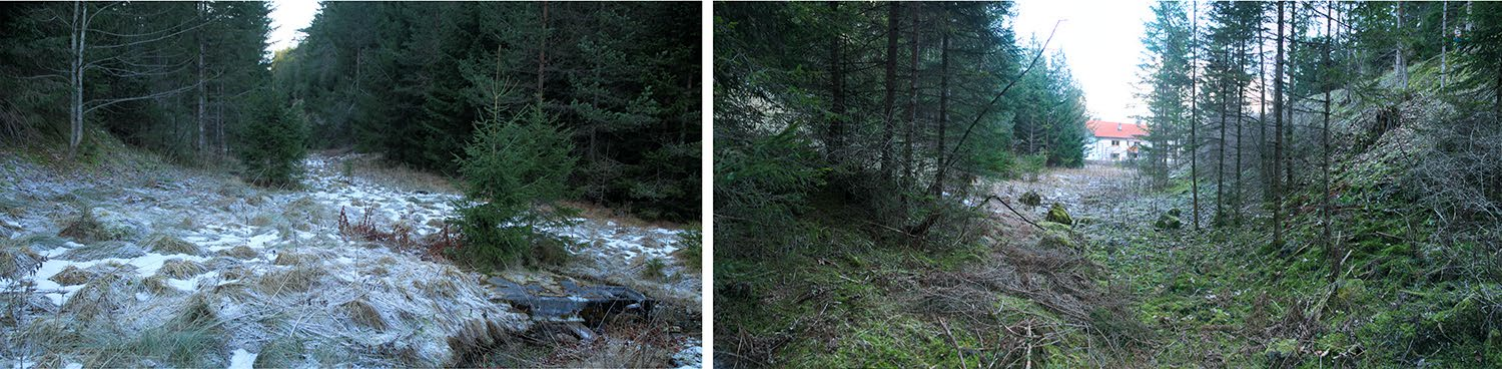
Views into the dry marginal valley from the east (left) and west (right). In the left image, the Dorfbach Spring can be seen in the lower right corner (images 2015-12-27).

Dorfbach, running west of Loisach, has a simpler course. Its three springs (QIT: “Brunnenquelle im Tal”: QU70803501; Fig. 8) are at the outlet of a dry valley south of estate Im Tal 15 at an altitude of 1005 masl (Fig. 9) on the northern slope of Narrenbichl at the base of a rock hillside with the local field name Alt Ristele (Plattenkalk limestone). From there, Dorfbach flows for 1377 m in a northeasterly direction until it reaches the Ehrwalder Moos plain at 968 masl. This results in a gradient of 26.9 ‰.

Hydrochemically, the water of the three springs clearly differs from the water of the Loisach; only downstream of the hotel Halali (between HAL and FBW in Table 2 and Fig. 1) do the results of a groundwater tracer test (green arrows in Fig. 4) and hydrochemical calculations show an influence of Loisach water, which seems to flow underground from the *Dreadlloch* (local language; spinning hole) swallow hole towards the Dorfbach. This hydro-chemical data shows that though both rivers are not identical, they come from a common stratigraphic source, proven by the Ca, Mg and hydrogencarbonate concentrations. Moreover, the flow data gives an indication of the different sizes of the two rivers, with the Loisach having on average a five times higher flow rate than the Dorfbach.

**Table 2:** Flow rates (*Q*) of the Dorfbach and Loisach Rivers measured with the salt tracer method in summer 2007 and August 2008.

Location	*Q*_2007_, L s^−1^	*Q*_2008_, L s^−1^	Location	*Q*_2007_, L s^−1^	*Q*_2008_, L s^−1^
**Dorfbach**			**Loisach**		
QIT	36	34	PFS	899	443
HAL	154	111	SOB	922	451
FWB	254	131	EWR	1265	697

Methodologically determined measurement errors of 5% (locations in Fig. 1).

Almost entirely, the bed of the Loisach lies either in the loose deposits of the Fernpass rockslide, in torrential fans or in fluvial fans (21–22 ‰ gradient). Just between the Narrenbichl and the power station, the river has cut into harder rocks of the Fernpass rockslide (79 ‰ gradient). In total, the gradient from the spring area to the Biberwier–Ehrwald road is 24 ‰. On the other hand, Dorfbach flows almost exclusively in a small valley between the deposits of the Fernpass rockslide and the Karnian to Norian Hauptdolomit dolomite and Plattenkalk limestone. Its gradient of 27 ‰ is in the same range as Loisach’s total gradient.

### Striking changes in the course of the Loisach river

**Figure 10:**
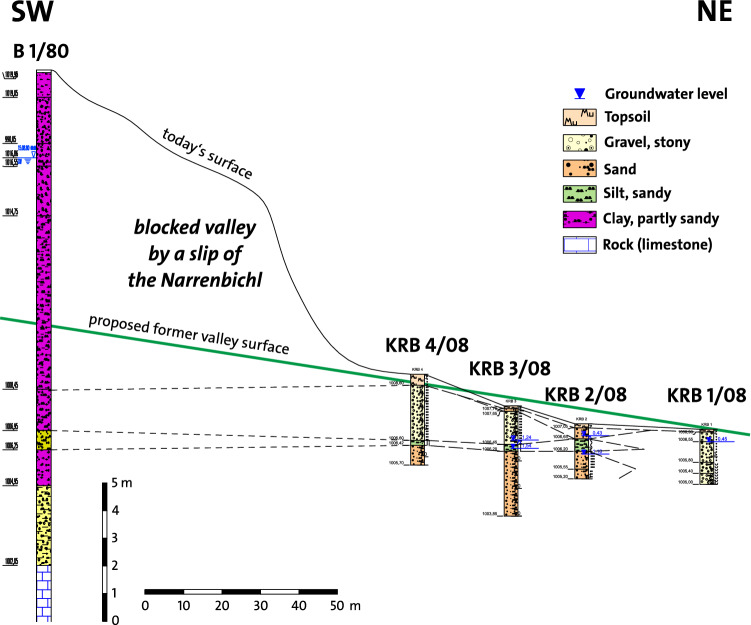
Results of drillings between the Falte Moos and the Dorfbach Spring (borehole 1/80 and RBK 1–4/08). Location of B 1/80 in Fig. 1. Symbols according to DIN 4023.

Three characteristics stand out in the course of the Loisach River (indicated with small numbers in Figs. 1 and 5): (1) the river bends abruptly to the southeast when reaching a former torrential fan at the Falte Moos, (2) a few hundred meters following the imaginary further course of the Loisach, a dry valley lines up with the Loisach course before its abrupt bend and (3) after approximately 500 m farther, the Dorfbach follows the direction of the dry valley. As with the step in the terrain on L391, the dry valley drops from 1020 to 1005 masl on less than a hundred meters (Fig. 9). At the dry valley entrance, Schuch^23^ has drilled through an 18 m thick sandy clay-silt sequence with its base at 1002 masl (borehole 1/80, Figs. 5 and 10). At the time of his drilling, the groundwater level was 1017 masl. Further exploratory drillings by the author found similar sand and silt in the dry valley and a groundwater level falling from 1008 to 1006 masl to the north, eventually emerging at the Dorfbach spring at 1005 masl (RKB 1–4 in Figs. 5, 8 and 10).

**Figure 11:**
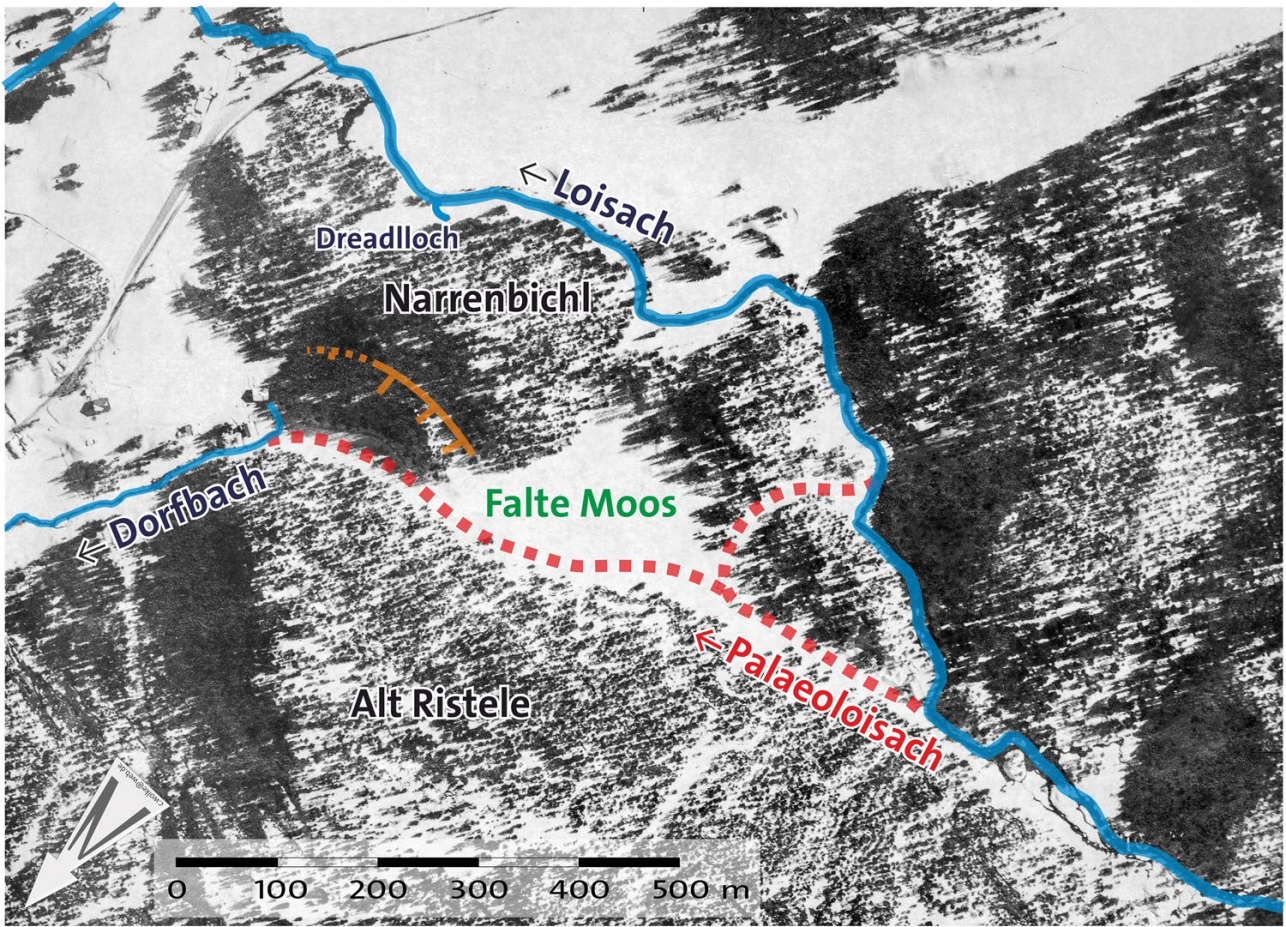
Reconstruction of the Paleoenvironmental conditions of the Palaeoloisach and Loisach in a March 1945 US aerial image (image 60–1060 of 1945-03-13). The edges in the terrain, indicating the slip of the Narrenbichl, and the Dreadlloch, are indicated (source: Luftbilddatenbank Dr. Carls GmbH/HES).

Locals believe that the Dorfbach spring is fed by the Loisach River through a groundwater connection from a swallow hole (ponor, locally called *Dreadlloch:* Figs. 1 and 11) at Sonnbichl road. However, despite three tracer tests with uranine (sodium fluorescein) in 2008, several flow measurements (Table 2) and a hydrogeochemical comparison of the waters, a connection from this swallow hole to the spring could not be proven. There is also no indication of flows through this swallow hole to the Dorfbach spring (QIT) when comparing the discharge before (PFS) and after (SOB) the swallow hole with the discharge of the Dorfbach spring. There was no substantial difference in the Dorfbach spring flow compared to the difference in the Loisach flow. If the flow through the *Dreadlloch* would influence the Dorfbach spring, a much larger influence of the flow would be expected. Slightly elevated Na^+^- and Cl^−^ concentrations, however, suggest that the Dorfbach spring is fed by groundwater from the dry valley, where a road salt silo of the Fernpass road is located. This has also been proven by hydrogeological investigations on Fernpass Road^23^.

A further bend in the course of the Loisach River is found in the Rauth district, where the river bends sharply from the northern direction into an eastern direction (Fig. 7). Between this point and the Dorfbach at the foot of the Neuwirtswände (Plattenkalk limestone), there is a relatively wide valley, which today is drained by a drainage ditch and of which the locals say that a lot of water was produced during construction. In fact, during drilling work (small boreholes to a maximum depth of 5 m, e.g., RKB S and RKB D in Fig. 5), a strong water flow in the Rauth area could already be demonstrated at a depth of 60 cm. Rounded and angular deposits of gravel and sand can be found at depths of up to 4 m, which can be interpreted as fluviatile deposits and possibly as basal till in the deeper boreholes, evidenced by difficult-to-drill, organic, grey–black sediments. Whether these are partly fluviatile sediments relocated by the Fernpass rockslide cannot be said without deeper drilling.

### Reasons for the Loisach’s diversion

Seven boreholes (Fig. 5), geological mapping results, Lidar images, aerial photographs before the construction of the Lermoos Tunnel and historical maps allow the reconstruction of Palaeoloisach history and especially the buried valley between the first sharp bend and the Dorfbach spring. The first maps date from the sixteenth century, and the first known aerial photograph is from 1942, while the first Lidar images date from 2006 to 2010 and were updated from 2012 to 2013.

Borehole data reveal that the Falte Moos is filled with approximately 18 m of a sandy silt–clay sequence interpreted as slope debris over basal till, indicating paleoenvironmental changes in the depositional conditions. The silt and sand layers could be glaciofluvial deposits, debris flow deposits and debris from the Fernpass rockslide. However, Abele^53^ mapped it as a “sedimentation zone” as well as a “debris flow or alluvial cone”, and the geological map at 1:50,000 indicated rockslide deposits. Using the detailed mapping of Abele in conjunction with the borehole data and additional field investigations, it becomes obvious that the Falte Moos is a marginal valley filled with sandy to loamy debris flow, alluvial and lake sediments having developed into a bogland which dewatered into a southerly direction towards the Loisach before it was modified by the Lermoos Tunnel construction works. In the dry valley, borehole data show sandy to silty loam, interpreted as fluvial deposits down to a depth of approximately 1002 masl. This depth corresponds to the base of the solid rock found in borehole 1/80.

**Figure 12:**
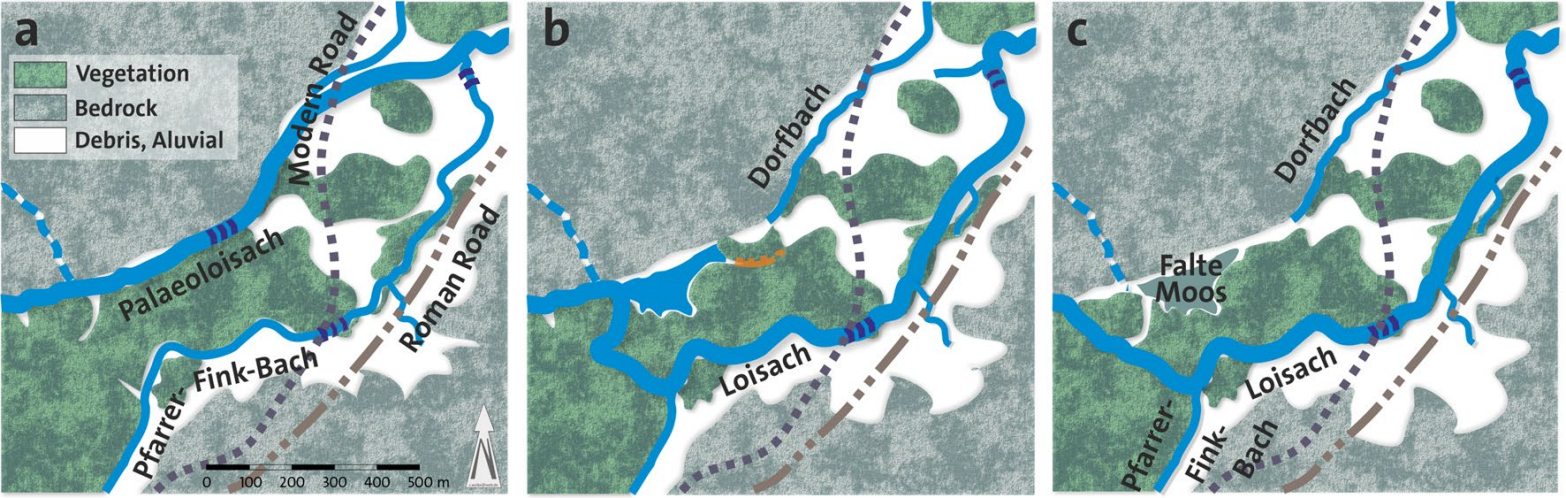
Reconstruction of the paleoenvironmental changes in the reach of the Palaeoloisach and Loisach. Indicated with double or triple arrows are the steps in the terrain in the Palaeoloisach and the Loisach described in the text. Course of the modern and Roman roads based on Walde and Grabherr^11^.

Prior to filling this marginal valley with sediments (Fig. 12a), the valley must have been blocked, the most likely location being the narrow between the Narrenbichl hill and the Neuwirtswände (Alt Ristele). As seen from the Lidar images, some aerial photographs, and field surveys, Narrenbichl has a sinus on its northern side, blocking the marginal valley, where Dorfbach emerges (Fig. 5). In addition, the Narrenbichl hill shows a small depression, indicating that the sinus results from a slip of the hill’s northern side into the marginal valley. This blocked the Palaeoloisach stream and consequently resulted in a blocked valley lake (Fig. 12b) and the filling of the marginal valley with lake and stream deposits, eventually resulting in the Falte Moos. Due to the filling, the Loisach was obstructed and had to find a new course, initially at the western end of the Falte Moos, later, as the lake filled, farther to the west, at its modern location (Fig. 12c).

### Contribution of the groundwater to the channel formation

Details about the formation of channels in the Fernpass rockslide deposits have been studied in a physical, analogue model by More and Wolkersdorfer^51^. This study originated from field observations during hydrogeological mapping in the Fernpass area, where “gurgling” of water could be heard in some of the depressions between the Toma hills. Therefore, a conceptional model was developed stating that internal erosion by groundwater caused the transport of finer material between the Toma hills and subsequent subsidence in these areas formed the channels between the Toma hills. In five physical laboratory experiments, this conceptual model was verified, and it could be shown that the cause for the channels in the Fernpass rockslide deposits is caused by internal erosion as a result from groundwater flowing predominantly in the weaker zones between the Toma hills.

## Dating the Palaeoloisach and the Narrenbichl slip event

### Evidence based dating

Dating the course of the Palaeoloisach is possible using the recession rate of the terrain step including probabilistic simulations as described below. In addition, using published studies and age determinations allows an age framing of the events described in this paper and to verify the results of the probabilistic method. Precise dating would require further and deeper core drilling with paleosol dating. For example, the 5 m deep drill hole (RKB D in Fig. 5) at the *Dreadlloch* showed a rooted paleosol soil at this depth possibly covered by a basal till. Whether it was from the Weißwände or from the Schachtkopf area cannot be judged without further investigation. To clarify this, drillings would be necessary in the area of the Falte Moos and in the Rauth area. Sarnthein^55^ mentions that the oldest deposits in lake Weißensee sediments date back to approximately 2000 BCE into the older Bronze age period. He concludes that this might have been the time when the first impermeable sediments formed within the troughs and consequently the marginal valleys. Abele^56^ reports a date of 305 ± 60 BCE for a spruce within a postrockslide deposit 5 km SW of Biberwier. According to the palynological data^10^, *sphagnum* mosses started to appear around 1000 BCE, indicating the existence of boggy areas. The next date that exists, is the construction of the Roman Via* Claudia Augusta* in 46 CE. Because it is know that the Roman engineers avoided wet areas, it can be concluded that the Falte Moos or the Falte Moos lake existed at that time. Finally, the Palaeoloisach must be older than the oldest known topographic maps, since they all show a Loisach with a bend to the southeast. Using the above age dating in the Fernpass area, the formation of the Palaeoloisach started shortly after the rockslide, around 2000 BCE and the Narrenbichl slip initiating the formation of the dry valley occurred in the late Subboreal to early Older Subatlantic between 1000 and 300 BCE (Table 3).

**Table 3:** Model parameters of the Monte Carlo simulation of the river Loisach recession rate at the terrain step.

Variable	unit	Model	*x*	*σ*	lower	upper	mode
Flow rate *Q*	m^3^ s^−1^	Normal truncated	0.7795	0.2882	0.4	1.3	–
Gradient *s*	–	Normal truncated	0.1	0.01	0.0732	0.1714	–
Stream width *W*	m	Normal truncated	1.5	0.05	1	2	–
Step height *H*	m	Discrete uniform	–	–	6	8.8	–
Density Water *ρ*	kg m^−3^	Normal truncated	999.862	0.1725	999.4223	1000.1053	–
UCS *q*_u_	N m^−2^	Pert	–	–	80 · 10^6^	180 · 10^6^	90 · 10^6^
Section length *L*	m	Discrete uniform	–	–	103	139	–
*k*	m a^−1^	Normal truncated	99.7	3	90	110	–
*n*	–	Normal truncated	0.73	0.015	0.69	0.77	–

*k* and *n* of the original publication were 99.7 and 0.73, respectively. UCS: unconfined compressible strength based on data from Kluckner^57^.

### Probabilistic dating

Waterfall or cliff recession rates have been used to date paleoearthquakes^58^ or landscape evolution^59,60^. These recession rates vary within a broad range from less than a millimeter to several meters annually, depending on various parameters such as type of waterfall, bedrock geology, rainfall intensity, climatic conditions, drainage area or bedrock channel concavity^58,61–65^. Therefore, a single recession rate value which disregards this variability can result in wrong extrapolations^66^. In general, arid regions, such as Turkey, show lower recession rates compared to humid areas such as Italy^61^, and waterfalls in softer rocks or sediments have higher recession rates than crystalline rocks^64^. Hayakawa and Matsukura^67^ developed an empirical equation that allows to calculate the recession rate based on the beforementioned parameters:

1 $$r_{\rm r} = k \cdot \left( {\frac{Q \cdot s}{{W \cdot H}} \cdot \sqrt {\frac{\rho }{{q_{\rm u} }}} } \right)^{n}$$

where *r*_r_ is the recession rate, *Q* the flow rate of the river, *s* the gradient within the knickpoint, *W* the river’s width, *H* the step hight, *ρ* the water density, *q*_u_ the unconfined compressible strength and *k* and *n* are factors determined by Hayakawa and Matsukura^67^ through regression of various waterfall data. To calculate the Narrenbichl slip event, the retrograde erosion rate of the terrain step was applied by using the recession rates calculated with Eq. 1. This follows the hypothesis that the steepening of the step occurred once the Palaeoloisach changed its course and replaced the substantially smaller Pfarrer-Fink-Bach (75 L min^−1^ in 2007). For calculating the time *T*_e_ since the Narrenbichl slip event occurred, the length of the terrain step *L* has to be divided by the recession rate *r*_r_:

2 $$T_{\text{e}} = \;\frac{L}{{r_{\rm r} }}$$

Because all variables in these equations are subject to variability, a probabilistic distribution method using Monte Carlo simulation with Simulación 5.0.5 (José Ricardo Varela) was applied, where *T*_e_—2022 provided the year of the event in BCE. Such an approach was also used by Lee et al.^60^ for the probabilistic dating of cliff recession, and it accounts for the problems using a single recession value. In addition to the seven river Loisach parameters, the two constants derived by Hayakawa and Matsukura^67^ were simulated within the error ranges of their regression line, adding up to nine fitting variables. All distribution models (Tab. 3) are based on measurements in the study area. To account for the high variability in the parameters, 10,000 simulation runs were needed to get statistically significant results.

**Figure 13:**
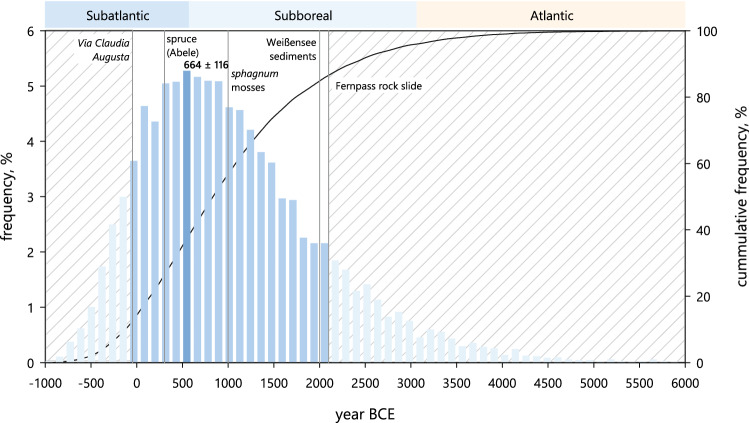
Probabilistic date distribution of the Narrenbichl slip and known datings in the Fernpass area (gray lines). Dates that are not possible due to the valley development are shown in lighter colours and in background shading. Most probable date (664 ± 116 BCE) of the Narrenbichl slip indicated in darker colour.

Grounded on this simulation (Fig. 13), the Narrenbichl slip occurred on the edge of the Older Subatlantic and Subboreal at 664 ± 116 BCE (later Bronze age). This fits into the evidence-based date, stating that the slip occurred in the Subboreal to early Older Subatlantic between 1000 and 300 BCE.

## Conclusions

Based on the morphological, geological and hydrogeological features of the terrain, it can be deduced that changing paleoenvironmental conditions affected the course of today’s Loisach. Shortly after the Fernpass rockslide 4100 ± 900 BP^28^ (≈ 2100 ± 900 BCE; “Fernpass cluster” *sensu* Prager et al.^27^), and the development of marginal valleys^51^, the Loisach flowed northwards in the orographically right (western) marginal valley between the rockslide masses, the Toma hills and the main dolomite and plate limestone (“Palaeoloisach”). This is indicated by a dry valley between the Narrenbichl and the Neuwirtswände (Alt Ristele), which seems too large to have been carved by the relatively small flow rate of the Dorfbach, even if one assumes a larger flow rate in the past (Fig. 11, Fig. 12). The step in the terrain at the Marienberg cable car lower station and the retrograde erosion of the limestone/dolostone and mudflow deposits there seem to be relatively young, indicating the relatively younger course of the Loisach and that an “older” course must have existed. During the Holocene climatic variations, periods of heavier rain, as discussed by Knapp et al.^9^, Huggel et al.^1^ or Prager et al.^27^, large-scale debris flows caused postrockslide paleoenvironmental changes of the Fernpass area. This further initiated a slip of the Narrenbichl, which blocked the valley and additional debris flow from the Weiß- and Neuwirtswände. Subsequently, “Loisach” debris filled the marginal valley and forced the Palaeoloisach into a new riverbed to the east, which essentially follows today’s course. Some of the water originating in the Weiß- and Neuwirtswände now flows as groundwater within these marginal valley fills towards the Dorfbach spring. A connection between the springs and the Loisach swallow hole (*Dreadlloch*) could not be proven by tracer tests or hydrochemically; rather, the increased Na^+^- and Cl^−^ concentrations in the Dorfbach Spring indicate an influence of road salt from the salt deposit at the Lermoos tunnel entrance. Only downstream of the hotel Halali, Loisach water seems to infiltrate as groundwater into Dorfbach, proven by tracer test results and the hydrochemistry of the Dorfbach water. An inactive undercut bank at the northernmost Toma hill in Biberwier suggests that the flow path of the Palaeoloisach, known as variant 1, is more likely than the flow path of variant 2 (Fig. 5), yet the centuries-long agricultural use of the valley blurred many additional traces of the Palaeoloisach. In variant 1, the Palaeoloisach flowed from the orographically right marginal valley through two Toma hills into the orographically left marginal valley. In variant 2, the Palaeoloisach would have followed the course of the current Dorfbach. However, since there is a relatively small cut into the sediments, this would be further evidence for variant 1 for the course of the Palaeoloisach. The current course of the Loisach beginning at the Falte Moos could correspond to an older course of the Pfarrer-Fink-Bach, which has its source in the area of the Untere Kohlstattboden.

Knowing the course of a Palaeoloisach might also explain the course of the Roman *Via Claudia Augusta* in the area of Biberwier (Fig. 12). *Via Claudia Augusta* stays in the middle of the furrow^12^^, p. 128^, while today’s road is more to the north-west. Should the Palaeoloisach once have occupied that area, it might be an explanation why the Roman engineers avoided that part of the furrow. Without doubt, this investigation is a contribution to understanding quaternary post rockslide developments occurring in this populated area and might help to interpret archaeological findings in the future.

Generally, it can be deduced that post rockslide development substantially changes the morphology and hydrogeological situation of the rockslide area and that changes in river courses might overprint important features for understanding the rockslide history itself. This can even result in partly misleading interpretations of the rockslide evolution itself as the post rockslide history becomes more prominent than the rockslide itself.

## Data Availability

On request, all relevant data can be obtained from the author. Historical map data and borehole data are available from the Tyrolean Government websites.
